# Evaluation of facial morphology and sagittal relationship between dental arches in primary and mixed dentition

**DOI:** 10.1590/2176-9451.20.4.063-067.oar

**Published:** 2015

**Authors:** Aline Traldi, Heloísa Cristina Valdrighi, Luciane Zanin de Souza, Silvia Amélia Scudeler Vedovello

**Affiliations:** 1 MSc in Orthodontics, Universidade de Araras (UNIARARAS), Araras, São Paulo, Brazil; 2 Professor, Universidade de Araras (UNIARARAS), Postgraduate program in Dentistry, Araras, São Paulo, Brazil

**Keywords:** Epidemiology, Dental occlusion, Primary dentition

## Abstract

**OBJECTIVE:**

To assess facial morphology (Pattern) and sagittal relationship between dental arches (Class), and establish a potential association between them and the variables sex, age and ethnicity, among schoolchildren aged between 4 and 9 years old (mean age of 6.7 years) in primary and mixed dentitions.

**METHODS:**

The sample comprised 875 children (457 males and 418 females) attending schools in Descalvado, São Paulo, Brazil. An attempt was made with a view to establish a potential association between children's morphological features with sex, age and ethnicity.

**RESULTS:**

Descriptive analysis revealed a predominance of facial Pattern I (69.9 %) and Class I (67.4 %). Statistical tests (*p* < 0.001) showed that Class I was more frequent among Pattern I children, whereas Class II prevailed among Pattern II, and Class III was frequent among Pattern I and III children. Ethnicity was the only variable associated with facial pattern.

**CONCLUSIONS:**

Results suggest that facial pattern and sagittal relationship between dental arches tend to be correlated. Ethnicity was associated with facial pattern, with Pattern I being the most recurrent among Caucasians and facial Pattern II being recurrent among Afro-descendant subjects.

## Abstract (pt)

**OBJETIVO:**

avaliar a morfologia facial (Padrão) e a relação sagital entre as arcadas dentárias (Classe) e determinar a associação entre sexo, idade e etnia, em escolares com 4 a 9 anos de idade (média de 6,7 anos), nas fases de dentadura decídua e mista.

**MÉTODOS:**

a amostra constou de 875 crianças (457 do sexo masculino e 418 do sexo feminino) de escolas do município de Descalvado/SP, que tiveram suas características morfológicas relacionadas ao sexo, idade e etnia.

**RESULTADOS:**

com base na análise descritiva dos dados, constatou-se que há predominância do Padrão I (69,9%) e da Classe I (67,4%). Nos testes estatísticos (p < 0,001), ficou evidente que a Classe I foi mais frequente no Padrão I; a Classe II, no Padrão II; e a Classe III, igualmente frequente nos Padrões I e III; apenas a raça teve associação com o padrão facial.

**CONCLUSÃO:**

o padrão facial e a relação sagital entre as arcadas dentárias tendem a estar relacionados, e a etnia tem associação com o padrão facial, sendo mais recorrente o Padrão I nos leucodermas e o Padrão II nos melanodermas.

## INTRODUCTION

Esthetics, facial beauty and excellence in occlusion are standards to be achieved in orthodontic treatment. Angle, in 1907,^1^ chose Apollo of Belvedere's face as ideal in terms of balance and beauty, and associated his face with the sagittal relationship established between dental arches. As a result, he came up with the sagittal classification of molar occlusion entitled "Class".

Since then, despite employing different methods, other authors^2^^,^ 3 ^,^ 4 have been concerned about establishing an association between occlusion and facial morphology, as there is strong evidence that such relationship is genetically determined. For this reason, it is important to identify how the face behaves in case of malocclusion, regardless of patient's age, since facial morphology is established at an early age.^5^

The spatial relationship established between mandible and maxilla tends to remain unchanged throughout the growth period, even though it has not achieved its final dimension at that point. This trend also applies to the sagittal relationship established between dental arches, with dental and facial patterns being determined at an early age. Thus, it is possible to reach diagnosis at the time of complete primary dentition.^6^^,^ 7

Clinical facial analysis reveals the spatial arrangement of basal bones, maxilla and mandible, and is capable of identifying facial balance or skeletal discrepancy. After this analysis is carried out, occlusion is assessed in an attempt to relate it to the facial skeleton.^4^

Clinical analysis of facial pattern is, therefore, important, considering that, according to the World Health Organization (WHO), malocclusion is ranked as the third most prevalent oral health problem in the overall population.^8^

In Brazil, data collected from "Projeto SBBrasil 2010" revealed a prevalence of 77.1% Class I, 16.6% Class II and 6.4% Class III malocclusions among 5-year-old patients.^9^ Since epidemiology is one of the pillars of public health, these population data are important to plan public health action, organize care services and recruit human resources.^10^

Once aware of facial pattern and occlusion, all that remains to understand is how the "Classes" behave in relation to each pattern. This allows coherent planning to be developed, since teeth implanted in bone bases tend to reflect the behavior of apical bases; thus, one would expect the following: Class I in Facial Pattern I, Class II in Facial Pattern II, and Class III in Facial Pattern III.^2^^,^ 3

Only a few studies have used facial analysis to establish correlations with the arrangement of teeth, especially in primary and mixed dentitions. Thus, this fact seems to be the rationale behind the present study. Therefore, the aim of this study was to assess facial morphology (Pattern) and sagittal relationship between dental arches (Class), and establish a potential association between them and the variables sex, age and ethnicity, among schoolchildren aged between 4 and 9 years old (mean age of 6.7 years) attending schools in Descalvado, São Paulo, Brazil.

## MATERIAL AND METHODS

This study was conducted upon approval of Uniararas Institutional Review Board (protocol #441/2011). The study population comprised schoolchildren aged between 4 and 9 years old, enrolled in 13 public schools and pre-schools and three private schools in the city of Descalvado, São Paulo, Brazil. 

In selecting the sample, the following inclusion criteria were applied: children who were in primary or mixed dentition with the presence of primary canines. Exclusion criteria were as follows: presence of orthodontic appliances and/or being subjected to previous orthodontic/orthopedic treatment, and children who were not authorized by their parents and/or guardians. Permission was given by means of signing an informed consent form.

Data were collected by means of clinical oral examination carried out by a previously calibrated professional. Data were analyzed by Kappa test which achieved a substantial score for acceptable parameters of reproducibility of examiner's methodology (0.86). 

In order to have facial pattern assessed, the children were taken to the school patio and instructed to remain standing up, looking straight ahead, in side view to the examiner, and with their head in natural position.^11^^,^ 12

Patients' sagittal clinical facial characteristics at rest and in lateral view were assessed based on the concept of pattern.^13^

Clinical examination allowed children's face to be classified as Pattern I, Pattern II and Pattern III. In Pattern I, there is facial balance between the maxilla and mandible, well positioned in relation to each other; there is proportion and balance between facial thirds, in addition to good zygomatic projection, pleasant nasolabial angle, passive lip seal or discrete interlabial space, well-determined mentocervical line and angle, and facial symmetry. Facial Pattern II is characterized by a positive sagittal step between maxilla and mandible, resulting from maxillary protrusion and/or mandibular deficiency, thus delineating a convex facial profile. Conversely, Facial Pattern III is characterized by a negative sagittal step between maxilla and mandible, resulting from mandibular prognathism and/or maxillary deficiency, thus delineating a concave or straight profile.^13^

Intraoral examination was also performed at the school patio, with children seated on ordinary chairs, under natural light, and with the aid of wooden spatulas. Interarch relationship was considered and determined by canine occlusion as Class I, Class II and Class III. Class I was determined when the tip of maxillary primary canine cusp occluded in the embrasure between mandibular primary canine and first molar, this being established as a case of normality. When maxillary primary canine is in mesial position, this relationship is established as Class II. When maxillary canine undergoes distalization, the relationship is established as Class III.^4^

Data were statistically assessed by descriptive analysis, and the associations established between independent (age, sex and ethnicity) and outcome variables (facial pattern and sagittal relationship between dental arches) were performed by means of chi-square test with significance level set at 5%.

## RESULTS

Sample comprised 875 children aged between 4 and 9 years old (mean age of 6.7 years), 418 of which were females (47.8%) while 457 were males (52.2%) of the following ethnic groups: Caucasian (71.1%), Afro-descendant (27.8%) and indigenous-descendant (1.1%).

The predominant facial pattern among children was Pattern I (69.9%), whereas the predominant sagittal relationship established between dental arches was Class I (67.4%).

When the associations established between independent (age, sex and ethnicity) and outcome variables (facial pattern and sagittal relationship between dental arches) were tested, only ethnicity was associated with facial pattern: Pattern I was recurrent among Caucasians while Pattern II was recurrent among Afro-descendants. There were insufficient indigenous-descendant patients to apply the association test.

There was statistically significant difference in the relationship established between dental arches and facial Pattern. Class I was more frequent in Facial Pattern I and less frequent in Facial Pattern III; Class II was more frequent in Facial Pattern II and less frequent in Facial Pattern III; and Class III was equally frequent in Facial Patterns I and III and less frequent in Facial Pattern II (*p* < 0.001; χ^2 ^= 283.060).

**Table 1.** Association between ethnicity and facial pattern* in children in complete primary and/or mixed dentition with the presence of primary canines.

Ethnicity	Pattern I	Pattern II	Pattern III	Total	*p*-value	
Caucasian	480	99	43	622	<0.001	70.491
Afro-descendant	124	103	16	243		
Total	604	202	59	865		

* There were insufficient indigenous-descendant patients to apply the test of association.

**Table 2.** Association between sagittal pattern (Class) and facial pattern in children in complete primary and/or mixed dentition with the presence of primary canines.

	Pattern I	Pattern II	Pattern III	Total	χ^2^	*p*-value
Class I	480	86	24	590	283.060	<0.001
Class II	100	107	3	210		
Class III	32	11	32	75		
Total	612	204	59	875		

## DISCUSSION

In view of the quest for facial esthetics, occlusal excellence and an increasingly preventive orthodontic approach, information acquired mainly by means of epidemiological survey is of great value in order to promote programs for malocclusion prevention, so that they come out in a lower degree of severity or are indeed prevented.^13^

Recognizing dental arch morphology, which is directly related to other parts of the craniofacial complex which all together determine the facial pattern of each individual, is of paramount importance for successful orthodontic treatment.^14^

The hurdles most frequently found are associated with scarcity of publications on this subject, which makes it difficult to establish preventive measures because, in each region, facial and occlusal alterations may vary widely. Moreover, the concept of beauty is not static, and undergoes changes over time and under influence of one's sex, cultural level, social values and media to a large extent, thereby varying widely among different populations.^15^

According to results yielded by Projeto SBBrasil 2010, there were no significant differences in the percentage of Class I canine relationship in Brazilian regions. However, there was lower prevalence of Class II canine relationship in the North in comparison to the South region of Brazil.^10^

In the present study, there were no statistically significant associations between age and occlusal relationship of dental arches (Class) and facial Pattern. However, in another study,^16^ this association was evident, since there was a higher prevalence of Class I and Class II in the age group ranging from 5 to 6 years old, and Class II in the age group ranging from 3 to 5 years old. This reduction in Class II in the age group ranging from 5 to 6 years old would be a consequence of children abandoning sucking habits in older age ranges. According to the literature, at the ages of 3 to 5 years old, there is a tendency towards sagittal relationship stabilty.^17^

Results yielded by the present study revealed that only ethnicity was associated with facial pattern. Pattern I prevailed among Caucasians, whereas Pattern II prevailed among Afro-descendants. It seems evident that morphogenesis influences facial architecture, since ethnicity present with specific features that differentiate one from the other. Afro-descendants present with greater bimaxillary protrusion in comparison to Caucasians, while indigenous-descendants present with an intermediate degree of protrusion between Caucasians and Afro-descendants.^5^

The sagittal relationship established between dental arches (Class) had no association with ethnicity; however, one study^18^ pointed out a high percentage of Class I (60%) among Caucasians, also showing a high prevalence of children with Class II, which is due to the high degree of miscegenation among the children evaluated.

In another study,^19^ Class II was more prevalent among Caucasians and Afro-descendants, whereas Class I prevailed among indigenous individuals; however, there was a high percentage of Class III among all ethnic groups. This may have occurred due to the high rates of tooth deterioration or loss, in addition to mesialization of first permanent molars. This consequence may be explained by lack of access to dental treatment. Importantly, it is paramount to be aware of such regional differences and epidemiological situations in order to be able to carry out planning and adequate orthodontic treatment for each population.

The results yielded by facial analysis in lateral view revealed a predominance of Pattern I (69.9%) in comparison to Pattern II (23.3%) and Pattern III (6.7%). Other authors^4^^,^ 20 have also pointed out this characteristic. These results are positive, given that the majority of children proved to have facial balance demonstrated by Pattern I and, in general, Pattern I children tend to grow with the same pattern and maintain it in skeletal maturity.

Facial patterns are classified as Pattern I, Pattern II, Pattern III, as well as long and short facial patterns. In this study, long and short facial patterns were not subject to analysis, particularly because they are difficult to diagnose in the age range considered herein. This is because children have not yet stopped growing up to the point of being able to characterize the face within these morphological types.^4^^,^ 6

Based on the concept of pattern,^13^ the face grows and maintains its configuration. Thus, it is possible to assess one's face since childhood, as from the time of complete primary dentition. Even though during primary and mixed dentition there is still a great deal of craniofacial growth, growth pattern deviations may already be detected and lead to the establishment of interceptive protocols in an attempt to adjust craniofacial growth.^4^

The present study highlights the predominance of Class I (67,43%) in comparison to Class II (24.00%) and Class III (8.57%). Various studies^4^^,^ 21 ^-^ 24 have assessed the sagittal relationship established between dental arches (Class); however, not all of them have used primary canine relationship, but have used permanent molar relationship instead. Nevertheless, the results yielded by the aforementioned studies are in agreement with the present survey, since Class I was most prevalent in all of them, followed by Class II and Class III. Conversely, in another study^25^ conducted in Pernambuco, Brazil, Class II (52.6%) was found to be more prevalent than Class I (36.8%) and Class III (10.5%). Importantly, it should be emphasized that, in this study, patients' clinical records were analyzed. These patients sought the Orthodontics and Facial Orthopedics postgraduate program clinic, seeking treatment due to presenting some type of malocclusion.

Clinically, one's occlusal relationship tends to reflect one's facial pattern. Nevertheless, this is not true in all cases, since one must consider the dentoalveolar compensations that may induce patterns of occlusal normality, even with the presence of deviations from normality of the pattern.^4^

Considering facial pattern as a primary etiological factor of malocclusion, Classes are reflections that characterize them.^13^ The results yielded in this research confirm this finding, since children with Class I sagittal relationship between dental arches had bone bases well related between them, which is determined as facial Pattern I (71%). Class II was more prevalent in Pattern II (51%), whereas Class III was equally frequent in Patterns I and III. Similar results were also obtained in another study,^4^ which allows us to conclude that sagittal occlusion conditions, the Classes, are influenced by genetically determined facial pattern.

In the majority of cases, teeth positioning is a consequence of the skeletal pattern that features a given malocclusion. Being aware of the relationship established between facial pattern and sagittal relationship between dental arches, in addition to the specific characteristics according to patient's ethnicity and sex, and early evaluations, enables clinicians to plan, determine the possibilities of treatment and, thus, achieve the best prognosis for each case.

## CONCLUSION

Facial pattern and sagittal relationship between dental arches are associated. Ethnicity was associated with facial pattern, with Pattern I being the most recurrent among Caucasians while Pattern II prevailed among Afro-descendants.
